# Integration of Oral Health into General Health Services for Older Adults

**DOI:** 10.3390/geriatrics8010020

**Published:** 2023-01-30

**Authors:** Alice Kit Ying Chan, Yiu Cheung Tsang, Chloe Meng Jiang, Katherine Chiu Man Leung, Edward Chin Man Lo, Chun Hung Chu

**Affiliations:** Faculty of Dentistry, The University of Hong Kong, Hong Kong, China

**Keywords:** older adult, elderly, oral health, prevention, caries, periodontal disease

## Abstract

The prevalence of oral diseases in the older adult population remains high worldwide and is expected to surge in the coming decade. The World Health Organization (WHO) has listed the oral health of older adults as one of its pivotal concerns. Oral health affects general health, and oral diseases increase mortality and morbidity in older adults. Integrating oral health into the general health service with a patient-centred approach can be an effective way to improve oral and systemic health for older adults simultaneously. This integration tackles the shared risk factors of both oral and noncommunicable diseases, aids in the early detection of systemic disease, strengthens health surveillance, enhances efficient data sharing, and allows for the better allocation of resources and the workforce in the healthcare system. However, the oral healthcare sector operates as an isolated field, with an emphasis on intervention rather than prevention, which presents a key challenge to the success of integration. Therefore, refocusing oral healthcare service on prevention is paramount. In addition, approaches taken in clinical practice implementation, interprofessional education and training, technology and innovation, research and evaluation, advocacy by national professional oral healthcare organizations, and policy making will ensure the efficient, effective, and long-term integration of oral and general health services. Integrating these services would foster the accessibility and affordability of oral healthcare services for older adults to improve their oral health and overall well-being in the coming decade. This review aims to discuss the merits and outline the challenges of integrating oral health into general health services for older adults and to propose the approaches that could be taken.

## 1. Background

Like all noncommunicable diseases, the prevalence of oral diseases increases throughout a person’s life due to their cumulative exposure to the various social and commercial determinants of oral health [1]. Therefore, older adults have a higher risk of contracting oral and systemic diseases compared to members of other age groups. Although oral diseases are mostly preventable, the rate of oral diseases in older adults remains unacceptably high [1]. Almost half of the global older adult population has untreated caries [2]. Two-thirds of the older adult population in the United States and Germany experience periodontitis [3,4]. A new definition of oral health developed by the FDI World Dental Federation General Assembly recognized the importance of oral health for people’s overall health and well-being [5]. Oral health is an integral part of healthy ageing and is associated with general health, morbidity, and mortality in older adults [6]. However, oral health inequities exist between and within countries, with a higher prevalence of oral diseases found in certain population groups, including patients with special needs, older adults, and people with low incomes [7]. Older adults may be cognitively impaired, functionally disabled, or medically compromised, making the provision of oral health services more challenging [8]. In addition, functional and physical disabilities act as barriers to dental access because extra services, such as walking sticks, wheelchairs, escorts, or transportation, are needed for assistance [8]. Dental treatment is often not covered in national health systems or insurance plans, making it unaffordable to older adults [9]. The United Nations has estimated that the global ageing population will double to 1.5 billion, with 1 in 6 older adults aged 65 or older by 2050 [10]. This will deepen the global burden of oral diseases in older adults and widen health inequalities. Oral healthcare professionals must develop ways to improve oral health, reduce oral health inequalities, and lessen the global burden of oral diseases in older adults by the next decade.

Due to the increasing population of older adults, the World Health Organization (WHO) has listed the oral health of older adults as one of its pivotal concerns [11]. Since both oral and noncommunicable diseases share a number of modifiable risk factors (e.g., smoking and diet) and social determinants, the WHO has suggested that integrating age-related oral health concerns into general healthcare may aid in the development of oral healthcare for older adults and help increase awareness of the importance of oral health, leading to improvements in the quality of healthcare for older adults [11]. The FDI World Dental Federation has indicated that integrated healthcare for older adults is the best approach to increase health outcomes and to tackle the profound consequences of population ageing in the next decade [12]. It has also listed “integrating oral care into general care” as one of the eight fields of action in the 2018 *Roadmap for Healthy Ageing* [13]. Integrated healthcare for older adults means that healthcare services extend across the span of care and are integrated within and among different professionals and sites of care within the healthcare system, including long-term care facilities and homes [12]. It does not mean merging the structures, but rather coordinating all healthcare professionals so that they can work together to provide a wide spectrum of services to older adults [12]. Furthermore, it aims to provide a comprehensive assessment for older adults, along with a common goal and a care plan that is shared among all healthcare professionals. Consequently, closely integrating oral health into the mainstream and general health services will not only reduce the global burden of oral diseases, but it will also promote good general health in older adults.

Integrating oral health into general health services is paramount for oral healthcare professionals to improve oral health in older adults in the coming decade. Oral healthcare professionals should identify the challenges they face and outline the approaches others can follow to ensure an efficient, effective, and long-term integration of oral and general health services for older adults. The objective of this review is to discuss the merits, outline the challenges, and propose approaches to integrating oral health into general health services for older adults. This review relied upon studies published in English, including reviews and communications, identified in the PubMed and Google Scholar databases, as well as the latest information available from the World Health Organization and FDI World Dental Federation General Assembly websites on the integration of oral health into general health services for older adults.

## 2. Merits of Integrating Oral Health into General Health Services for Older Adults

### 2.1. Promoting Primary Healthcare by Controlling Shared Risk Factors

Oral diseases commonly accompany other noncommunicable diseases that share the same risk factors and social and commercial determinants [14]. However, these noncommunicable diseases can be prevented by promoting oral health in older adults and by controlling the associated risk factors through dietary advice, smoking cessation, and moderating alcohol consumption [15]. Dental caries are caused by the destruction of hard dental tissues through acidic byproducts from the bacterial fermentation of free sugars [16]. The consumption of free sugars is one of the risk factors for root caries that is commonly found in older adults [2], and it is also a risk factor for obesity and diabetes [17]. Dietary advice on the reduction of free-sugar intake provided by oral healthcare professionals can aid in preventing dental caries, reducing overweight or obesity rates, and controlling diabetes [17]. Oral healthcare professionals can provide further advice on proper nutritional intake for older adults to help shape healthier lifestyles. Smoking has been linked to periodontal disease and cancer of the mouth and throat [18], and it is a risk factor for coronary heart diseases, respiratory diseases, stroke, and cancer of the lung, pancreas, kidney, and urinary tract [15]. Oral healthcare professionals play a pivotal role in promoting tobacco cessation to improve both oral and general health for older adults. High levels of alcohol consumption increase people’s risk of oral cancer and other noncommunicable diseases, such as high blood pressure, cirrhosis of the liver, and cardiovascular diseases. It is also linked to head trauma, including jaw and tooth fractures [15]. Advice on alcohol consumption from oral healthcare professionals may reduce the incidence of these oral and systemic diseases. Integrating oral healthcare into general health services can push oral healthcare professionals into the mainstream of overall healthcare programmes to promote both oral and general health. Health promotion should focus on the whole population rather than on disease-specific at-risk groups, and it should be performed in a less costly but more effective and efficient way [15].

### 2.2. Early Detection of Systemic Diseases

Older adults have a higher chance of developing multiple chronic medical conditions, and some chronic systemic disorders may go undetected during their early stages [8]. However, some may present early signs and symptoms in the form of ulcers, white and red patches, halitosis, gingival bleeding, or dry mouth in the oral cavity [19]. Diabetic patients may have poor periodontal conditions that present with gingival bleeding and clinical attachment loss [19]. Patients with leukaemia may present with mucosal bleeding, ulceration, and localized or diffused gingival enlargement [19]. Oral healthcare professionals may detect these medical conditions during their early stages through oral screening and can collect saliva samples as biomarkers to detect systemic diseases [20]. Saliva collection is rapid, simple, and noninvasive [20], and it helps to quickly detect diabetes mellitus, cardiovascular diseases, pancreatic cancer, breast cancer, lung cancer, and prostate cancer, as well as to provide appropriate interventions to reduce the rate of mortality and morbidity in the later stages of these diseases [20]. This allows oral healthcare professionals to collaborate with medical colleagues in screening and detecting systemic disorders in older adults before they develop into serious medical problems.

### 2.3. Improving Systemic Health

Oral health is related to general health, mortality, and morbidity [6]. The bidirectional relationship between periodontal diseases and type 2 diabetes mellitus is well-established [21]. Chronic progressive diseases, such as periodontal disease and other noncommunicable diseases, release inflammatory mediators into the circulation and exacerbate systemic inflammation [22]. This shared inflammatory pathway is believed to be an association between periodontal disease and some systemic diseases, including cardiovascular diseases, rheumatoid arthritis, Alzheimer disease, and Parkinson disease [8]. Aspiration of oral bacteria causes pneumonia, especially in hospitalized patients and older adults [22]. If dental caries or periodontal disease are left untreated, bacteria from the cavities or periodontal pockets may enter the bloodstream, leading to sepsis [23]. Therefore, oral health promotion in older adults can improve both oral and systemic health.

### 2.4. Assisting in Health Surveillance and Enhancing Data Sharing

The dental profession can access ageing populations through regular dental check-ups or community services to collect data on oral health status, behavioural risk factors, medical history, and if resources are available, biochemical information, such as *Streptococcus mutans* levels and salivary or serum markers for oral health surveillance [24]. Since oral diseases and noncommunicable diseases share common risk factors, these data can also facilitate the surveillance of chronic systemic diseases [25]. Integrating oral health into general health services will enhance data sharing among different specialities. For example, oral healthcare professionals could access patients’ medical and drug history in preparation for a dental visit to ensure older adults are treated in a proper and safe way. In addition, medical teams could identify the oral conditions that are affecting a patient’s current medical condition and provide appropriate advice and treatment. Data from dental and medical practices can also be merged efficiently for research purposes to report the prevalence and severity of oral and systemic diseases or to measure the impact of interventions in improving ageing populations’ health.

### 2.5. Better Allocation of Resources and Workforce in the Overall Healthcare System

After integration, promotion of both oral and general healthcare can be performed through a population-wide approach rather than a disease-specific approach. Preventive programmes can be developed in a more cost-effective manner by addressing the shared, modifiable risk factors and social and commercial determinants of both oral and systemic diseases, which would be particularly beneficial in resource-constrained countries [15]. Having oral healthcare professionals in healthcare communities will allow them to acquire the knowledge and skills necessary to be redeployed in other roles within health systems alongside other health professionals in case of unforeseen events, such as pandemics and other catastrophic disasters. Dental professionals have knowledge of and skill with clinical practice and implementation of infection control, which was an invaluable resource in the recent COVID-19 pandemic response [26]. They have shouldered some of the front-line clinical duties, including screening suspected cases, conducting swabbing operations, delivering immunization vaccines, and providing consultations on infection control measures in many countries [26]. Their contributions proved that an integrated healthcare system can be more resilient if it has an extra workforce for clinical duties.

## 3. Challenges of Integrating Oral Health into General Health Services for Older Adults

The 2008 *World Health Report on Primary Health Care* recommended that the healthcare system, including the oral health sector, should be integrated into a multidisciplinary care pathway to increase the effectiveness of care for patients with special needs, such as elders or patients with cognitive or physical disabilities [27]. The integrated healthcare system would provide universal access to a wide range of health services to reduce health inequities among societies [27]. However, this integrated approach remains in the earliest stage in many countries and has faced resistance from some disciplines, such as the oral healthcare sector [28], due to challenges in policy making, education and work culture, and administration.

The oral healthcare sector operates as a field isolated from mainstream healthcare, and it is financed differently from general healthcare; therefore, the oral health of the population always receives lower priority within national policy planning [29]. Oral healthcare is not part of universal healthcare coverage and is not even included in health benefit packages in many countries [29]. In most countries, dentistry still adopts intervention-oriented and dentist-centred approaches rather than prevention-oriented approaches to manage oral diseases [30]. Many dental schools train their dental students to work in isolation and rarely train other skilled healthcare professionals to address the oral health needs of the global population [30]. Unlike in medicine, primary healthcare is not prioritized in dentistry, and dentists usually work alone rather than in a team. Differences in education and working culture, along with unfamiliarity with other ways of working, create additional barriers to integration.

Healthcare systems in most countries are organized in a disconnected and fragmented way, with poor referral systems, insufficient connections among different professional sectors, the use of different systems in recordkeeping, and a lack of coordinated staff [28], which make it difficult to implement the integration. Regarding these challenges, reforms will inevitably be required and sustained through the efforts of all healthcare professionals, academic leaders, groups of healthcare professionals, and policy makers to integrate oral health into general health services for older adults.

## 4. Approaches to Integrating Oral Health into General Health Services for Older Adults

### 4.1. Clinical Practice

Multidisciplinary clinical settings with both dental and medical practices in place can facilitate interdisciplinary collaboration as referrals and communication can be achieved easily [28]. The collaborative process can be further smoothened with the aid of coordinating staff [28]. The multidisciplinary team should meet regularly to share information, define clinical roles, and evaluate the effectiveness of the integration [12]. Contemporary dental practices should shift the mode of oral care from the traditionally intervention-oriented one to a prevention-oriented one, and they should focus on controlling the shared, modifiable risk factors and the social and commercial determinants [30]. This can be achieved through risk assessment, dietary advice, and tobacco cessation. Oral healthcare professionals should raise older adults’ awareness about general health issues, such as the relationships between oral health and diabetes, obesity, cardiovascular diseases, and other chronic medical conditions, and they should have a comprehensive risk assessment. Tailor-made dietary advice should be provided, not only to prevent caries, but also to moderate overweight and diabetes and to promote healthy eating habits to obtain adequate nutrition in older adults. Oral healthcare professionals should encourage tobacco cessation for older adult smokers by implementing the 5A model (ask, advise, assess, assist, and arrange), arranging behavioural counselling, using nicotine replacement therapy, and giving proper referrals to their national smoking cessation institution if needed [31]. Oral healthcare professionals should also form a closer link with medical colleagues to detect and intervene in chronic medical conditions quickly by observing the early oral manifestations and by collecting saliva samples as biomarkers for initial screening.

### 4.2. Education and Training

In universities, education should be available for all related healthcare professionals, including dental, medical, nursing, and social science schools. Basic knowledge of geriatric medicine and dentistry should include common medical and oral problems and ways to manage them in older adults, special precautions for delivering healthcare to older adults, and skills for assisting their daily living activities. The curricula should also aim to develop skills in interdisciplinary collaboration and communication. For instance, the dental curriculum should be evidence-based, prevention-oriented, and patient-centred, rather than intervention-focused.

For integration, a lack of interprofessional education and training lowers healthcare professionals’ competency [28]. Therefore, oral and other healthcare professionals should be encouraged to attend conferences beyond their specialties to broaden their knowledge of and skill in other professions. Participating in regular seminars and interdisciplinary case discussions can help stimulate knowledge exchange among interprofessional teams.

### 4.3. Technology and Innovation

Information and communication technologies improve access, quality, safety, and, most importantly, the integration of healthcare services [12]. The use of integrated practice-management software systems allows professionals to enter, share, and merge electronic patient records from oral and other healthcare professionals. This shared data system is easily understandable and encourages interprofessional communication, and the integrated data can be utilized to monitor and evaluate patient needs. Automated reminders or warnings from this health software system can help healthcare professionals deliver service in a more effective and safer way, especially with different special precautions to manage older adults’ medical conditions.

### 4.4. Research and Evaluation

Oral healthcare will be more empowering and evidence-based in the next decade [32]. Therefore, it is imperative to provide evidence that supports oral health policy and its integration into general healthcare services. More research should be conducted to investigate the link between oral health, noncommunicable diseases, and frailty, and to evaluate the health economics of integrated healthcare systems to support revisions to oral health remuneration systems that facilitate integration with general health services. Data collection for research purposes requires a reliable monitoring and surveillance system that uses a set of common oral health indicators. Oral healthcare professionals should collect common oral health indicators regularly and record them in a surveillance network that is shared with other healthcare professionals. These data can be utilized to monitor the oral health conditions of older adults and to evaluate the effectiveness of the integrated health services and the implemented preventions and interventions for oral diseases in older adults.

### 4.5. Advocacy by National Professional Oral Healthcare Organizations

Professional oral healthcare organizations should promote oral health outside of dentistry by offering seminars or activities in other professional healthcare organizations to raise awareness on the importance of oral health and its relationship to general health. They should also conduct hands-on activities with geriatric medicine groups for interprofessional skill-training to improve their competency in providing oral and general healthcare services to older adults. Recently, the European Federation of Periodontology organized interdisciplinary awareness campaigns, such as “The Perio and Cardio Project” together with World Heart Federation and the “Perio and Diabetes” campaign. These campaigns promoted the awareness of the links between periodontal and systemic diseases and provided specific guidelines for oral health professionals, medical professionals, patients, and the public. Such activities are essential to promoting interdisciplinary collaboration. In addition, professional oral healthcare organizations can advocate for oral health, not only addressing the most common oral diseases, such as caries and periodontal disease, but also discussing some of the more devastating oral conditions, such as oral infections and oral cancer, which is more prevalent in old age, to demonstrate the similarities between oral and systemic health conditions. The national oral healthcare professional organizations should bear the responsibility for advocating to prioritise oral health and its integration into general health services for older adults in policy agendas and to support and aid in all-sugar-free policies and government tobacco-cessation programmes.

### 4.6. Policy Making

The success of any proposed program relies on support from higher-level policies, financial aid, and shared responsibility among teams [12]. The government should implement universal health coverage policies to provide accessible and affordable healthcare services, both oral and general, to all people, including disadvantaged groups, such as older adults [32]. It should also prioritize the overall healthcare of older adults, and it should design strategies to integrate oral health into general health, beginning with controlling their shared risk factors. Oral healthcare should be included in general health coverage or reimbursement schemes to make it more affordable for older adults and more feasible to integrate it with general health services. Furthermore, the government should implement population-wide health policies to enhance awareness of common risk factors for oral diseases and noncommunicable diseases, and it should conduct health programmes in communities, elderly daycare centres, and nursing homes to promote oral and general health in older adults. For instance, the government in Hong Kong launched the “Outreach Dental Care Programme” to provide basic dental care in residential homes for older adults. The programme also offers oral care training to caregivers in long-term care facilities and delivers oral health education talks to older adults and their family members to enhance their oral health knowledge [9]. Also, the national healthcare model should be reshaped to focus on patient-centred care, with all stakeholders being integrated to improve both oral and general health in ageing populations.

The commercial determinants of oral and noncommunicable diseases can only be effectively tackled by government policies and legislation [30]. Policies to reduce sugar consumption and increase tobacco cessation should be enforced and strengthened. The taxation of sugar-sweetened beverages and other sugary products and of tobacco is effective at controlling the risk factors [32]. The power and influence of the global sugar and tobacco industries can be further limited by implementing strong regulations and legislation. For instance, clear and transparent guidelines for conflicts of interest should be established to deflect their influence away from academic research, health policies, and professional healthcare organizations.

Countries vary in cultural background and social structures and differ in their available resources and workforce. Therefore, the approaches recommended above may not be effective in all countries or regions. They should be selected and adjusted according to individual countries’ circumstances. Policy makers, international healthcare professional organizations, national dental associations, public health professionals, researchers, academics, and individual healthcare professionals should all have a common goal to integrate oral health into general health services to provide patient-centred healthcare to ageing populations so as to improve their oral health and overall well-being in the coming decade [12]. All individual oral healthcare professionals should be prepared to bear more vital, leading roles within the healthcare community to improve both oral and general health in older adult populations.

## 5. Conclusions

The burden of oral diseases remains high in older adult populations worldwide, and it is expected to surge in the coming decade. Oral health is related to general health, mortality, and morbidity in older adults. Refocusing healthcare services on prevention is paramount. An effective way to improve oral health in older adults is to integrate oral health with general health services to tackle the shared risk factors and provide effective management for oral diseases through a patient-centred approach over the next decade. This integration will aid in the reallocation of resources and the workforce in the healthcare system and make oral healthcare more accessible and affordable to all, including disadvantaged groups such as older adults.

## Data Availability

No new data were created or analyzed in this study. Data sharing is not applicable to this article.
